# Ventral Pallidal GABAergic Neurons Drive Consumption in Male, But Not Female, Rats

**DOI:** 10.1523/ENEURO.0245-24.2025

**Published:** 2025-02-03

**Authors:** Alexandra Scott, Anika Paulson, Collin Prill, Klaiten Kermoade, Bailey Newell, Elizabeth A. Eckenwiler, Julia C. Lemos, Jocelyn M. Richard

**Affiliations:** ^1^Graduate Program in Neuroscience, University of Minnesota, Minneapolis, Minnesota; ^2^Medical Discovery Team on Addiction, University of Minnesota, Minneapolis, Minnesota; ^3^Department of Neuroscience, University of Minnesota, Minneapolis, Minnesota; ^4^Molecular Pharmacological and Therapeutics, University of Minnesota, Minneapolis, Minnesota

**Keywords:** chemogenetic, consumption, sex differences, sucrose, ventral pallidum

## Abstract

Food intake is controlled by multiple converging signals: hormonal signals that provide information about energy homeostasis and hedonic and motivational aspects of food and food cues that can drive nonhomeostatic or “hedonic” feeding. The ventral pallidum (VP) is a brain region implicated in the hedonic and motivational impact of food and food cues, as well as consumption of rewards. Disinhibition of VP neurons has been shown to generate intense hyperphagia, or overconsumption. While VP GABA neurons have been implicated in cue-elicited reward-seeking and motivation, the role of these neurons in the hyperphagia resulting from VP activation remains unclear. Here, we used designer receptors exclusively activated by designer drugs to activate VP GABA neurons in nonrestricted male and female rats during chow and sucrose consumption. We found that activation of VP GABA neurons increases consumption of chow and sucrose in male rats, but not female rats. Together, these findings suggest that activation of VP GABA neurons can stimulate consumption of routine or highly palatable rewards selectively in male rats.

## Significance Statement

The ventral pallidum (VP) has been implicated bidirectionally in consumption of both standard food and highly palatable rewards, but the specific neural subpopulations involved have not been identified. Here, we chemogenetically excited GABAergic ventral pallidal neurons and tested consumption of standard chow and a sweet sucrose solution. We found that chemogenetic excitation of these neurons stimulated consumption of both rewards but did so specifically in male rats. These results suggest that GABAergic ventral pallidal neurons can drive overconsumption of foods in male rats, but not female rats, raising important questions about the role of VP in consumption in females, who have been understudied in this domain.

## Introduction

Food intake is controlled by multiple converging signals: the primary attributes of food (i.e., taste and smell), environmental cues that attract individuals to the food, and hormonal signals that provide information about energy homeostasis. Neural circuits integrate these cues and signals to initiate food-seeking and consumption (Morton et al., 2014). Excessive cue reactivity can lead to eating beyond the caloric need for energy homeostasis. The ventral pallidum (VP) is implicated in controlling both food consumption and cue-elicited reward-seeking. Disinhibition of the VP increases food intake in rats (Stratford and Kelley, 1999; Stratford and Wirtshafter, 2013), especially food high in fat (Covelo et al., 2014). The VP is also an essential node in the neural circuitry that underlies motivation to food/reward-seek. VP neurons are known to respond to cues predictive of reward, and VP neuronal responses encode the motivational value of these cues (Tindell et al., 2005; Smith et al., 2011; Ahrens et al., 2016; Richard et al., 2016, 2018). Recent research has begun to probe which VP neuronal cell types contribute to consumption and reward-seeking. The VP contains three major neuronal subtypes: cholinergic, glutamatergic, and gamma-aminobutyric acidergic (GABAergic). VP glutamatergic neurons are excited by aversive stimuli and constrain reward-seeking (Faget et al., 2018; Tooley et al., 2018). In contrast, many VP GABA neurons are excited by rewards and pavlovian reward-paired cues (Stephenson-Jones et al., 2019). Recent work indicates that VP GABA neurons encode the incentive motivational value of cues and causally contribute to cue-elicited reward-seeking (Scott et al., 2023), yet it remains unclear whether these same neurons are responsible for the hyperphagia produced by hyperactivation of the VP.

Recent work has found mixed effects of chemogenetic manipulations of VP GABA neurons on food-seeking and consumption (Farrell et al., 2021; Li et al., 2021). Chemogenetic inhibition of VP GABA neurons decreases food-seeking (i.e., operant responding) of chow and palatable food (banana pellets) in food-restricted rats (Farrell et al., 2021), whereas inhibition of VP GABA neurons in sated rats reduces palatable food-seeking but not chow-seeking behavior. In contrast, inhibition of VP GABA neurons fails to significantly reduce nonoperant consumption of palatable food in nonrestricted rats (Farrell et al., 2021). Prior work also found that activation of VP GABA neurons does not increase consumption of chow in food-restricted mice (Li et al., 2021). Together, these findings might suggest that VP GABA neurons control food-seeking but not consumption, which would be surprising considering the prior work discussed above (Cromwell and Berridge, 1993; Stratford and Wirtshafter, 2013; Covelo et al., 2014). Yet, the internal state of animals is known to impact VP GABA responses to reward and reward-seeking (Stephenson-Jones et al., 2020; Farrell et al., 2021, 2022). Additionally, VP manipulations have been shown to have differential influence over different macronutrients (Covelo et al., 2014). Overall, it is still unclear how activation of VP GABA neurons in nonrestricted animals affects consumption of home chow or rewards of single macronutrient origin. Furthermore, the prior work has not disaggregated testing subjects by sex. Here, we used chemogenetic methods to test whether VP GABA neuron activation affects consumption of chow or 10% liquid sucrose in nonrestricted male and female rats. We found that VP GABA neuron activation alters consumption in a sex-biased manner.

## Materials and Methods

### Subjects

Male and female Long–Evans rats (Envigo), weighing 250–275 grams at arrival, were individually housed in a temperature- and humidity-controlled colony room on a 14/10 h light/dark cycle. All experimental procedures were approved by the Institutional Animal Care and Use Committee at our institution and were carried out in accordance with the guidelines on animal care and use of the National Institutes of Health of the United States.

### Surgeries

During surgery, rats were anesthetized with isoflurane (5%) and placed in a stereotaxic apparatus, after which surgical anesthesia was maintained with isoflurane (0.5–2.0%). Rats received preoperative injections of carprofen (5 mg/kg) for analgesia and cefazolin (70 mg/kg) to prevent infection. Syringes for viral delivery were aimed at the ventral pallidum (VP) using the following coordinates in comparison with bregma: +0.3 mm AP, ±2.3 mm ML, −8.3 mm DV. To achieve cell-type–specific expression of designer receptors exclusively by designer drugs (DREADDs), 0.60–0.80 µl of a 1:1 mixture of AAV8-GAD1-cre (8.29 × 10^13^; University of Minnesota (UMN) Viral Vector and Cloning Core; Scott et al., 2023) and cre-dependent DREADD virus was injected bilaterally into VP. Gq DREADD virus (*n* = 23, 12 males, 11 females: AAV8-hSyn-DIO-hM3Dq-mCherry, ≥4 × 10^12^ vg/ml, Addgene) was mixed with GAD1-cre to express excitatory designer receptors in VP GABA neurons. Gi DREADD virus (*n* = 15, seven males, eight females: AAV8-hSyn-DIO-hM4Di-mCherry, ≥1 × 10^13^ vg/ml, Addgene) was mixed with GAD1-cre to express inhibitory designer receptors in VP GABA neurons. For external controls (*n* = 17, nine males, eight females), 0.6–0.8 µl of a 1:1 mixture of GAD1-cre and mCherry virus (AAV8-hSyn-DIO-mCherry, ≥1 × 10^13^ vg/ml; Addgene) was injected bilaterally into VP. Virus was delivered to VP through 33-gauge injectors at a rate of 0.1 µl per min, for a final injection of 0.6–0.8 µl per side. Injectors were left in place for 10 min following the infusion to allow virus to diffuse away from the infusion site. Rats recovered for at least 1 week before beginning handling. Rats recovered for at least 4 weeks before training to allow time for sufficient viral expression.

### DREADD ligand and administration

Injections of DREADD ligand were administered on test days through intraperitoneal (IP) injection. DREADD ligand, JHU37160 (J60: HB6261, Hello Bio; Bonaventura et al., 2019), was dissolved in saline and diluted to a concentration of 0.05 and 0.50 mg/ml. Ligands were injected at 1 ml/kg of volume to body weight. We selected these doses based on prior work showing behavioral effects of 0.01–1 mg/kg in rats and mice expressing hM3Dq (Bonaventura et al., 2019), with submaximal effects occurring at the lowest dose. For ex vivo electrophysiology, we tested a concentration of 10 µM based on prior work (Huang et al., 2021).

### Behavioral testing

#### Chow consumption testing

Rats were habituated to the testing environment 3 d before DREADD ligand or saline injection test days. The testing environment consisted of individually housed cages with corn cob pellet bedding and water. On test days, chow (Teklad Global 18% Protein Rodent Diet, pellets, 2818, Envigo) was premeasured at 20 g for each subject. On separate test days, counterbalanced for order, rats received IP injections of 0, 0.05, or 0.5 mg/kg J60 and were immediately placed in test cages. One group (*n* = 28) had the chow placed into the cage at the time of injection, meaning that these animals had an hour and a half for consumption. Another group (*n* = 34) received chow 30 min postinjection, meaning that these animals had an hour for consumption. Ninety minutes after the injections, the final amount of chow was recorded, and the amount consumed was calculated. Observation days followed each test day to ensure that the DREADD ligand did not have any lingering consequences on behavior. On these days, food was preweighed as on test days, rats were placed in the chambers without receiving any injections, and food was weighed again 90 min later. We observed no effects of ligand injections on consumption on the observation days.

#### Sucrose consumption testing

Ten percent sucrose was initially provided to subjects overnight prior to the start of the experiment to reduce neophobia. Rats were then habituated to the testing environment 3 d before DREADD ligand or saline injection test days. The testing environment consisted of individually housed cages with corn cob pellet bedding and a two-bottle top with water and sucrose bottles. Bottle placement was balanced across subjects. On test days, water and sucrose bottles were weighed before the test. After IP injection was given, rats were immediately placed in the testing chambers. One group (*n* = 15) received water and sucrose availability at the time of injection, meaning that the animals had access to water and sucrose for an hour and a half. Another group (*n* = 22) received access to water and sucrose 30 min postinjection, meaning the animals had access to water and sucrose for an hour. The amount of water and sucrose was recorded after 90 min, and the amount consumed was calculated. Observation days followed each test day to ensure that the DREADD ligand did not have any lasting impacts on behavior. On these days, rats did not receive injections and had access to sucrose bottles for the same amount of time as on test days. Injection order was counterbalanced across animals. Sucrose preference was calculated as the difference between the amount of sucrose consumed and the amount of water consumed, divided by the total amount of liquid consumed.

### Histology

#### Region-specific DREADD expression validation

To confirm that DREADD virus was expressed in VP, 40 µm sections were imaged on a wide-field fluorescence microscope (Olympus MVX10). We verified the location of DREADD expression in all animals (*n* = 49) based on anatomical markers of VP (anterior commissure, lateral ventricle size). Seven animals were excluded for lack of bilateral DREADD expression. To quantify the regional selectivity of DREADD virus expression, we further conducted detailed examination of tissue from the VP as well as the regions anterior to the VP (+0.72 to 2.28 mm ahead of bregma) and posterior to the VP (−0.72 to −1.72 mm behind bregma) of a subset of rats (*n* = 8; *n* = 4, two males, two females of 600 nl viral injections; *n* = 4, two males, two females of 800 nl viral injections). All images were acquired with the same acquisition settings. The QUINT workflow (EBRAINS) was then used to assess the 3D spread of virus expression. Briefly, the 2D images of each section from each animal were matched to atlas (Waxholm Space Atlas v4) segmentations in the QuickNII software. Then, masks of mCherry expression, of each section/animal, were made in the ilastik software, with labels differentiating between background and expression. Finally, quantification of regional expression was calculated with atlas segmentations and masks of sections in the Nutil software. The final output contained all atlas brain regions in our sections and the amount of mCherry expression (pixel quantification) in each region.

#### DREADD functional validation: c-Fos staining

Rats from each DREADD group were administered either 0, 0.05, or 0.50 mg/kg J60 in saline solution 1 h prior to perfusion. Rats were deeply anesthetized with pentobarbital and perfused intracardially with 0.9% saline followed by 4% paraformaldehyde. Brains were removed, postfixed in 4% paraformaldehyde for 4–24 h, cryoprotected in 20% sucrose for >48 h, and sectioned at 40 µm on a microtome for c-Fos immunofluorescence. Briefly, sections were washed in PBS, blocked in normal donkey serum, and incubated in the c-Fos primary antibody (1:2,500 rabbit anti-c-Fos, Cell Signaling Technology, 2250S) overnight at room temperature. Sections were then washed in PBS and incubated in the fluorescent-conjugated secondary antibody (1:250 Alexa 488 donkey anti-rabbit, Invitrogen catalog #A-21206) for 2 h at room temperature. Sections were washed in PBS, wet-mounted on coated glass slides in PBS, air-dried, and coverslipped with Vectashield mounting medium with DAPI. Imaging of the tissue was done on a confocal microscope (Nikon A1R, University Imaging Centers) at 20× with 2× digital zoom. The exact same acquisition settings were used on all tissue samples. HALO software (Indica Labs) was used to quantify the amount of DREADD-expressing cells (mCherry-positive cells) that colocalized with the early activation gene, c-Fos. Briefly, the total cell number and location within an image were determined by nuclear staining (DAPI). mCherry and c-Fos were assigned their respective fluorophores within the software, and the software compared the location of each fluorophore to the location of the DAPI-labeled cells to confirm fluorophore expression is associated with a self-defined nucleus and cell size. HALO analysis software and software settings were optimized for the tissue from all animals, and then settings were used to batch analyze cell counts of mCherry- and c-Fos-positive cells.

#### DREADD functional validation: ex vivo electrophysiology

Preparation of VP slices was optimized for VP health (Matsui and Alvarez, 2018). Rats expressing mCherry or Gi DREADD were anesthetized with pentobarbital, perfused intracardially with warm ACSF (33°C), and decapitated. Brains were rapidly extracted, and 240 µm slices were cut in carbogenated, warm ACSF with kynurenic acid (3 mM) on a vibratome (Leica VT1200S). Slices were incubated in carbogenated, warm aCSF with kynurenic acid (3 mM) for 30 min and then moved to room temperature. Recordings were conducted at physiological temperature (31–33°C) in ACSF with NBQX (5 µM), CPP (5 µM), gabazine (5 µM), and CGP 55845 (2 µM) to isolate autonomous activity. Recording pipettes (3–6 MΩ resistance) were filled with K-gluconate internal containing 1% biocytin. Recordings were performed in cell-attached voltage-clamp configuration holding at 0 mV. After gigaΩ seals were made, cells were allowed to acclimate for 5 min before baseline recordings began. Spontaneous activity was monitored for 5 min at baseline, and for 10 min with J60 (10 µM) washed on. Data were acquired at 5 kHz and filtered at 1 kHz using Double IPA (Sutter Instrument). Data were analyzed using pClamp (Molecular Devices). The solutions contained the following (in mM): for ACSF, 124 NaCl, 2.5 KCl, 2.5 CaCl_2_, 1.3 MgCl_2_, 26.2 NaHCO_3_, 1 NaH_2_PO_4_, and 20 glucose (310–320 mOsm); for K-gluconate internal, 130 K-gluconate, 5 NaCl, 10 HEPES, 0.2 K-EGTA, 1 MgCl_2_, 10 phosphocreatine, 5 Mg-ATP, and 0.5 Na-GTP (pH = 7.25, 290 mOsm).

### Statistical analysis

Statistical significance was assessed by fitting a linear mixed model to examine the effects of the designer drugs with fixed effects for sex, ligand dose, and virus and a random effect of rats. When significant main effects or interactions were found, pairwise comparisons were run with Sidak corrections for multiple comparisons. Quantification of DREADD expression spread was also assessed by fitting a linear mixed model to examine the effects of injection volume on viral spread and expression of DREADD virus. Linear mixed models were fit with fixed effects for sex, volume, and brain region, and a random effect of rat, as well as a random effect of cell, where appropriate.

## Results

**Figure 1.**
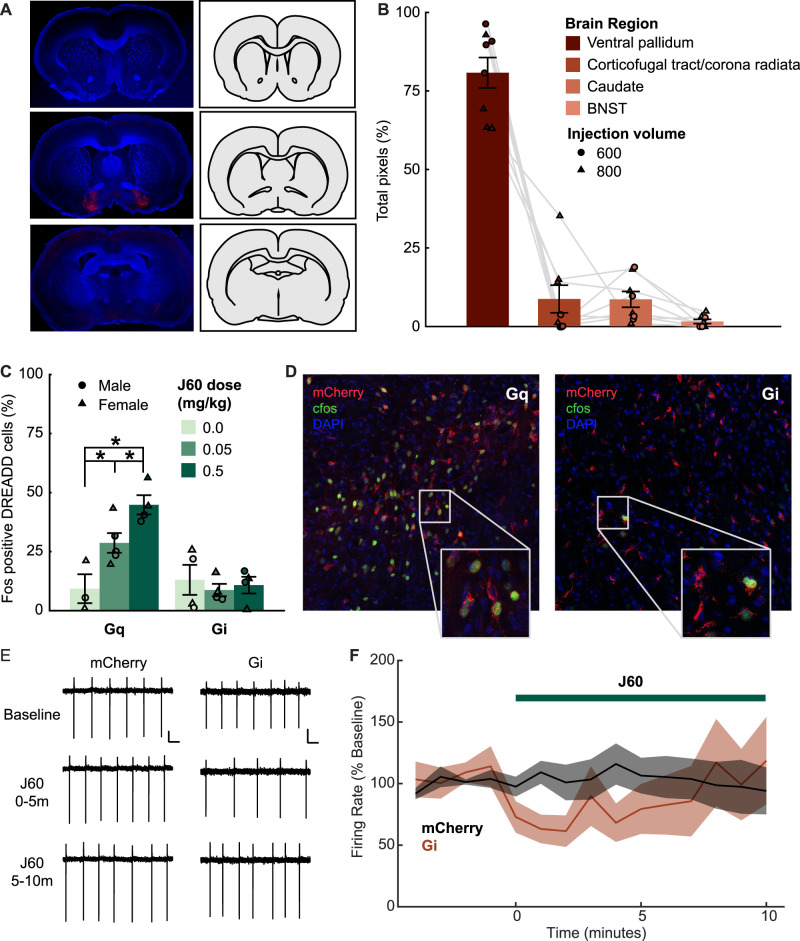
Characterization of DREADD localization and functional impact. ***A*** Representative section from the striatum (top), ventral pallidum (middle), and septum (bottom), used for analysis in QUINT workflow. ***B***, Output results of QUINT workflow (*n* = 8), showing all brain regions where mCherry (DREADD virus reporter) was detected in rats that received 600 nl (*n* = 4, circles) and 800 nl (*n* = 4, triangles) injections. LME showed a significant effect of brain region (*F*_(3,28)_ = 113.34, *p* < 0.001), on viral particles, with follow-up comparisons indicating that significantly more expression was found in VP compared with other brain regions where expression was seen. ***C***, Percent of DREADD-positive cells expressing c-Fos after 0, 0.05, or 0.5 mg/kg J60 in male (circles) and female (triangles) rats expressing Gq or Gi DREADD. *p < 0.05 (left). ***D***, Representative VP images from a Gq male (left) and a Gi male (right) injected with 0.5 mg/kg J60. ***E***, Representative traces from neurons expressing mCherry (left) or Gi DREADD (right) during the baseline period and 0–5 and 5–10 min after J60 application. ***F***, Normalized firing rate (% of baseline) of neurons expressing mCherry (black; 9 cells/3 rats) and Gi DREADD (red; 15 cells/3 rats) at baseline and following J60 wash-on (0–10 min). Lines and shading indicate mean ± SEM.

Here we assessed the impact of chemogenetic manipulation of VP GABA neurons on sucrose and chow consumption in *ad libitum* fed male and female rats. In addition to verifying that all included rats had bilateral DREADD virus expression centered in VP, we conducted a more detailed analysis of eight randomly selected Gi and Gq brains to determine the extent of viral expression spread to adjacent brain regions (Fig. 1*A*,*B*). Most fluorescence (81.2 ± 4.86% of objects, a proxy for cell count) was restricted to the VP. We then identified additional atlas regions outside VP in which more than one rat had >5% of their fluorescent object counts. We observed expression exceeding this benchmark in three of eight rats in the corticofugal tract and corona radiata (8.72 ± 4.41% mean ± SEM), and in four of eight rats in the caudate (8.60 ± 2.52%). The next region with the most extra-VP expression was the bed nucleus of the stria terminalis (BNST; 1.51 ± 0.67%), in which no rats had >5% of their fluorescent objects. Overall, we did not observe substantial expression in all rats in any specific brain region other than the VP. These results suggest that our behavioral findings are the result of chemogenetic manipulation of VP GABA neuron activity, and not due to changes in activity in adjacent brain structures. We also assessed the functional impact of the DREADD manipulations by assessing c-Fos expression (Fig. 1*C*,*D*). In rats expressing Gq DREADD, we found that both doses of J60 significantly increased the proportion of mCherry cells expressing c-Fos (Fig. 1*C*,*D*; main effect of ligand, *F*_(2,6)_ = 11.45, *p* = 0.0089). Importantly for the interpretation of the behavioral results described below, sex had no effect on c-Fos expression (main effect of sex, *F*_(1,6)_ = 0.41, *p* = 0.55), and we observed no interaction between sex and the effect of the ligand (sex by ligand interaction, *F*_(2,6)_ = 0.12, *p* = 0.89). Furthermore, when we assessed the impact of J60 in Gq females alone, we found a significant effect of ligand (*F*_(2,3)_ = 27.59, *p* = 0.011), despite the null behavioral results reported below. We observed no effect of ligand on c-Fos expression in rats expressing Gi DREADD (main effect of ligand, *F*_(2,6)_ = 0.18, *p* = 0.83), and no effect of sex (*F*_(1,6)_ = 0.02, *p* = 0.88) or interaction of sex and ligand (*F*_(2,6)_ = 0.52, *p* = 0.62). This is likely due to low basal levels of c-Fos under control conditions. To further assess the functional impact of J60 activation of the Gi DREADD, we conducted cell-attached voltage-clamp recordings of spontaneous activity in VP GABA neurons expressing mCherry or Gi DREADD, during a 5 min baseline period and for 10 min after washing on 10 µM J60 (Fig. 1*E*,*F*). While we found a trend toward an interaction between bin and viral group when we compared a 5 min baseline to the first 5 min after J60 was applied (*F*_(1,44)_ = 2.23, *p* = 0.14), this was not the case when we compared the baseline to subsequent 5 min period (*F*_(1,42)_ = 0.0097, *p* = 0.92) or when we assessed all three bins (*F*_(1,66)_ = 0.004, *p* = 0.94), suggesting that any cells that are inhibited initially by J60 application may be accommodating over time. We also observed no significant main effects of time independently of the viral group. Altogether these results do not suggest robust inhibition of VP GABA neurons by J60 acting on Gi DREADD receptors on timescales relevant to our behavioral testing. Because both functional validation experiments of the Gi DREADD were inconclusive, we have omitted any Gi behavioral results here.

### Activation of VP GABA neurons affects consumption of sucrose in a sex-biased manner

**Figure 2.**
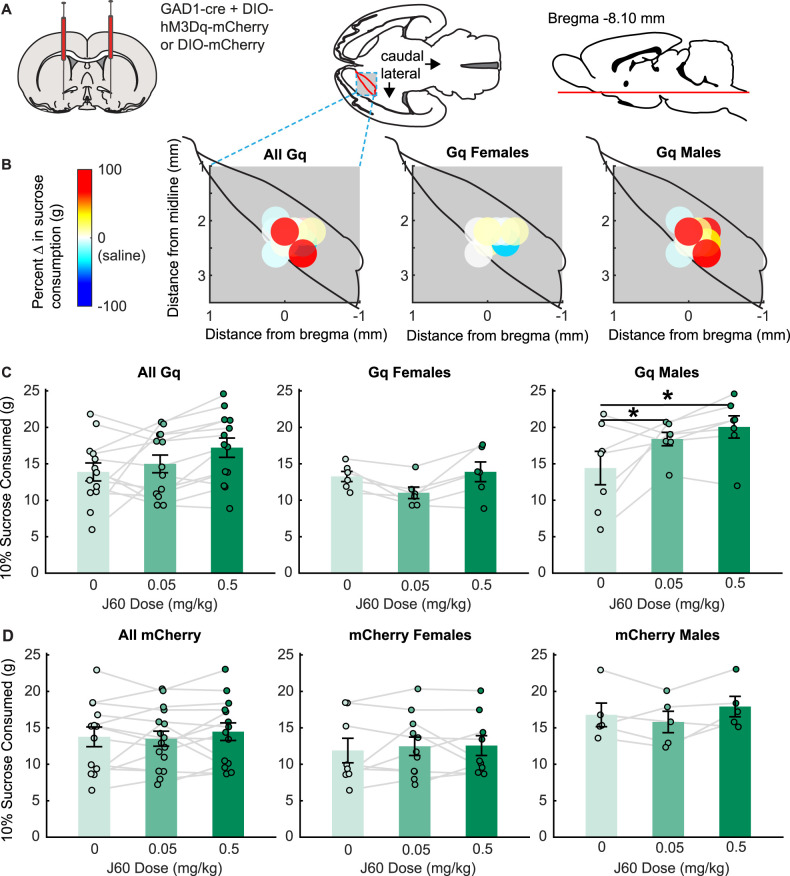
Effects of chemogenetic activation of VP GABA neurons on sucrose consumption. ***A***, A mixture of GAD1-cre and DIO-hM3Dq-mCherry or DIO-mCherry was injected bilaterally into the VP. ***B***, Colormaps show hM3Dq viral placement (brightest point of expression) in a horizontal cross section of the VP and behavioral changes from baseline following a high dose (0.5 mg/kg) of J60, for all Gq (hM3Dq) rats (left), female Gq rats (middle), and male Gq rats (right). ***C***, Sucrose consumption following injections of saline (light green), low-dose J60 (medium green), and high-dose J60 (dark green) for all Gq rats (left), Gq females (middle), and Gq males (right). ***D***, Sucrose consumption following injections of saline (light green), low-dose J60 (medium green), and high-dose J60 (dark green) for all mCherry control rats (left), mCherry females (middle), and mCherry males (right). Bar plots and error bars indicate mean ± SEM. Dots and lines represent individual rats. Asterisks indicate *p* < 0.05 for Sidak-corrected pairwise comparisons.

When we examined the impact of Gq DREADD chemogenetic activation of VP GABA neurons on sucrose consumption (*n* = 13, six females, seven males; Fig. 2*B*,*C*), we found effects that varied by sex. In rats expressing the Gq DREADD, we found significant effects of ligand dose (*F*_(2,22)_ = 4.27, *p *= 0.03) and sex (*F*_(1,11)_ = 9.60, *p *= 0.01) and a ligand by sex interaction (*F*_(2,22)_ = 4.40, *p *= 0.03). Follow-up comparisons indicated that both low and high doses of J60 DREADD ligand increased sucrose consumption in male Gq rats relative to saline control. We found no effects of the J60 ligand in female Gq rats. In contrast, when we assessed sucrose consumption in mCherry control vector rats (*n* = 13, eight females, five males; Fig. 2*D*), we found no significant effect of ligand dose (*F*_(2,25)_ = 1.18, *p* = 0.32) or sex (*F*_(1,12)_ = 4.45, *p* = 0.06) and no ligand by sex interaction (*F*_(2,25)_ = 1.78, *p* = 0.19). Furthermore, when we directly compared sucrose consumption and preference in Gq and control rats, we found a significant interaction between sex, ligand injection, and virus (*F*_(2,47.14)_ = 5.06, *p* = 0.01). When we split these data by sex, we found a trend toward an interaction between ligand and virus in male rats (*F*_(2,20)_ = 2.8337, *p* = 0.082; sucrose) and a significant interaction between ligand and virus in female rats (*F*_(2,27.11)_ = 3.50, = 0.04), though the latter interaction was not explained by any significant pairwise comparisons. When we excluded the high dose from the model (due to nonspecific effects observed in the chow test below), we continued to find the same interaction patterns. Given prior work suggesting that VP plasticity is altered in “weight gainers” (Gendelis et al., 2020), we also assessed a linear mixed model that included weight as an additional factor. We found that the inclusion of weight did not improve our model fit (AIC without weight, 430.46; AIC with weight, 437.08), and that weight did not have a main effect on sucrose consumption (*F*_(1,26.11) _= 0.08, *p* = 0.77). We also did not observe any interactions between rat weight and ligand, or rat weight, ligand, and virus (*F* values = 0.15–0.88). Overall, these results indicate that chemogenetic activation of VP GABA neurons increases consumption of sucrose selectively in male rats, suggesting that VP GABA neurons impact consumption in a sex-biased manner.

### Activation of VP GABA neurons impacts sucrose preference in a sex-dependent manner

**Figure 3.**
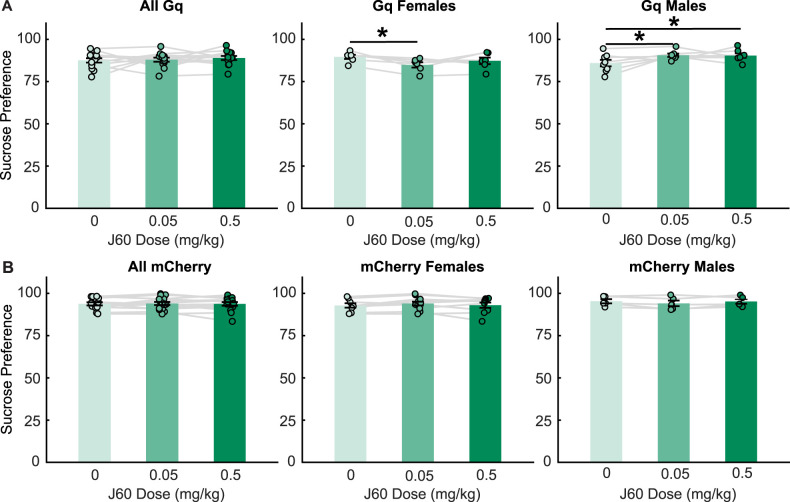
Effects of chemogenetic activation of VP GABA neurons on sucrose preference. ***A***, Sucrose preference following injections of saline (light green), low-dose J60 (medium green), and high-dose J60 (dark green) for all Gq rats (left), Gq females (middle), and Gq males (right). ***B***, Sucrose preference following injections of saline (light green), low-dose J60 (medium green), and high-dose J60 (dark green) for all mCherry control rats (left), mCherry females (middle), and mCherry males (right). Bar plots and error bars indicate mean ± SEM. Dots and lines represent individual rats. Asterisks indicate *p* < 0.05 for Sidak-corrected pairwise comparisons.

Sucrose preference was measured as the percentage of total liquid solution consumed (sum of water and sucrose solutions) that was sucrose solution, during the consumption test. All included rats were noted to have a baseline sucrose preference on habituation days. When male and females were pooled, Gq rats tested for sucrose preference (*n* = 13, six females, seven males) did not differ in their preference for sucrose across saline (87.51% ± 1.28), low ligand (87.95%  ± 1.19), or high ligand (88.95% ± 1.20) test days (Fig. 3*A*). No significant effect of ligand dose (*F*_(2, 22.97) _= 0.39, *p* = 0.68) or sex (*F*_(1, 10.90)_ = 1.16, *p* = 0.31) was observed in a linear mixed-effects model. However, a ligand by sex interaction (*F*_(2, 22.97)_ = 6.30, *p* = 0.007) was observed in the model. Follow-up comparisons revealed that low doses of ligand significantly (*p* = 0.03) decreased sucrose preference in female rats, whereas both low and high doses of ligand significantly (*p* = 0.02, *p* = 0.03) increased sucrose preference in male rats. Additionally, sucrose preference following injections of low-dose J60 was significantly different between male and female rats (*p* = 0.02). This effect is aligned with sucrose consumption findings where males consumed more following both doses of ligand and females did not significantly change their sucrose consumption but tended to decrease their consumption compared with vehicle control. In comparison, in control mCherry rats, we found no significant effect of ligand dose (*F*_(2, 25.03)_ = 0.13, *p* = 0.98), sex (*F*_(1, 11.99)_ = 0.61, *p* = 0.45), or ligand by sex interaction (*F*_(2, 25.03)_ = 1.79, *p* = 0.19) for sucrose preference (Fig. 3*B*). Importantly, when we directly compared Gq and mCherry rats more directly, we found a significant interaction between ligand injection, sex, and virus (*F*_(2,48.05)_ = 7.47, *p* = 0.0015). When we split the data by sex, we found a significant interaction between injection and virus in female rats (*F*_(2,27.07)_ = 5.93, *p* = 0.0073) and a trend toward an interaction between injection and virus in male rats (*F*_(2,20.973)_ = 2.73, *p* = 0.088). When we excluded the high dose from the model, we still found an interaction between ligand injection, sex, and virus (*F*_(1,25.12)_ = 19.98, *p* < 0.001) and significant interactions between ligand injection and virus in both male rats (*F*_(1,10.93)_ = 5.59, *p* = 0.038) and in female rats (*F*_(1,14.123)_ = 30.9143, *p* < 0.001). As for sucrose consumption, we also assessed a linear mixed model that included weight as a factor. We found that the inclusion of weight did not improve our model fit (AIC without weight, −299.21; AIC with weight, −290.49), and that weight did not have a main effect on sucrose preference (*F*_(1,23.26)_ = 0.22, *p* = 0.64). We also did not observe any interactions between rat weight and ligand, or rat weight, ligand, and virus (*F* values = 0.23–1.07). Overall, these results suggest that Gq activation of VP GABA neurons has opposing effects on sucrose preference in male and female rats.

### Activation of VP GABA neurons did not impact water consumption

In Gq rats tested for sucrose consumption and preference, water intake did not differ across saline (1.89 ± 0.24), low dose (1.94 ± 0.16), and high dose (2.05 ± 0.21) test days. No significant effects of injection (*F*_(2,22)_ = 0.21, *p* = 0.81), sex (*F*_(1,11)_ = 0.71, *p* = 0.42), or injection by sex interaction (*F*_(2,22)_ = 0.62, *p* = 0.54) were found in a linear mixed-effects model. When we compared Gq and mCherry control rats, we did not find any interactions between virus and other factors (*F* values = 0.05–1.06).

### Sex-biased effects on standard chow consumption may be driven in part by nonspecific effects of the ligand

**Figure 4.**
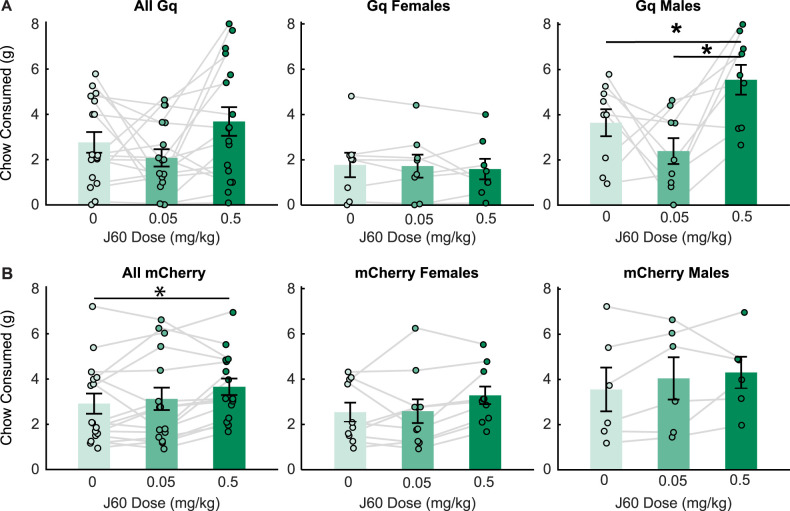
Effects of chemogenetic activation of VP GABA neurons on chow consumption. ***A***, Chow consumption following injections of saline (light green), low-dose J60 (medium green), and high-dose J60 (dark green) for all Gq rats (left), Gq females (middle), and Gq males (right). ***B***, Chow consumption following injections of saline (light green), low-dose J60 (medium green), and high-dose J60 (dark green) for all mCherry control rats (left), mCherry females (middle), and mCherry males (right). Bar plots and error bars indicate mean ± SEM. Dots and lines represent individual rats. Asterisks indicate *p* < 0.05 for Sidak-corrected pairwise comparisons.

When we examined the impact of Gq DREADD chemogenetic activation of VP GABA neurons on chow consumption, we also found effects that varied by sex (Fig. 4). In Gq rats tested with chow (*n* = 17, eight females, nine males; Fig. 4*A*), we found a significant effect of ligand dose (*F*_(2, 19.49)_ = 5.09, *p* = 0.01), sex (*F*_(1, 26.19)_ = 13.68, *p* = 0.002), sex by ligand interaction (*F*_(2, 23.42)_ = 6.12, *p* = 0.006). Pairwise comparisons indicated that high-dose J60 increased chow consumption in males relative to the saline condition and the low dose condition. In contrast, the J60 DREADD ligand had no effect on consumption in female Gq rats. Unexpectedly, when we examined mCherry control vector rats tested for chow consumption (*n* = 16, 10 females, six males; Fig. 4*B*), we found a significant main effect of ligand dose (*F*_(2,28)_ = 3.56, *p* = 0.04), but no significant effect of sex (*F*_(1,14)_ = 1.97, *p* = 0.18) or ligand by sex interaction (*F*_(2,28)_ = 0.41, *p* = 0.67). Follow-up comparisons revealed that a high dose of ligand significantly (*p* = 0.01) increased chow consumption in controls, independent of sex, compared with saline. There was no significant effect of low dose (*p* = 0.44) compared with saline, or significant differences between ligand doses (*p* = 0.06). This suggests that J60 may act nonselectively at higher doses, consistent with some recent reports (Van Savage and Avegno, 2023). Importantly when we directly compared Gq and control rats, we found a significant interaction between sex, ligand injection, and virus (*F*_(2,58)_ = 5.44, *p* = 0.0068). When we compared the impact of J60 on chow consumption in male Gq versus control rats, we found a trend toward an interaction between ligand and virus (*F*_(2,26)_ = 3.22, *p* = 0.056). Yet, when we excluded the high dose from our analysis, we did not find a significant interaction between ligand and virus in male rats (*F*_(1,13)_ = 1.94, *p* = 0.18). In female rats, we also found a trend toward an interaction between virus and ligand (*F*_(2,32)_ = 3.03, *p* = 0.06) when all doses were included, but no trend when the high dose was excluded (*F*_(1,16)_ = 0.043, *p* = 0.84). We also assessed a linear mixed model that included weight as a factor. Interestingly, while the addition of weight improved the model (AIC without weight, 370.07; AIC with weight, 356.65), we did not find a main effect of weight on chow consumption (*F*_(1,28.69)_ = 0.023, *p* = 0.88), or any interactions of rat weight and ligand or rat weight, ligand, and virus (F values = 0.63–1.94). Overall, we did not observe any significant effects of the low dose of J60, which elevated sucrose consumption and preference in male Gq rats, and reduced sucrose preference in female Gq rats. While we observed heightened chow consumption in male Gq rats after the high dose of J60, these effects may be explained, at least in part, by the nonselective effects of the ligand.

## Discussion

Here, we assessed how chemogenetic activation of VP GABA neurons impacts consumption of 10% sucrose solution and regular chow in *ad libitum* fed rats. Chemogenetic activation of VP GABA neurons with a low dose of the J60 ligand increased sucrose consumption and preference in male rats. In contrast, chemogenetic activation of VP GABA neurons with this same dose in female rats failed to increase sucrose consumption, and even reduced sucrose preference, suggesting sex-biased effects of GABA neuron activation. The effects of VP GABA neuron activation may be selective to palatable rewards, such as sucrose, as we did not observe any significant effects of low-dose J60 on chow consumption in male or female rats. The high dose of ligand increased chow consumption in male rats expressing the Gq DREADD, as well as sucrose consumption and preference, but we also observed increases in chow consumption in response to high-dose J60 in control animals, regardless of sex. Overall, these results suggest a sex-biased role for VP GABA neurons in consumption, which may selectively promote consumption of palatable rewards.

### Activation of VP neurons impacts consumption in a sex-specific manner

When we activated VP GABA neurons in *ad libitum* fed male rats, we observed increases in sucrose consumption and preference at both doses of ligand, and increases in chow consumption at the high dose of ligand, which also altered chow consumption in controls. This effect differs from the findings in Li et al. (2021), where activation of VP GABA neurons had no significant effect on food intake in male mice. Importantly, this prior study tested chow consumption in food-restricted mice, so their null finding may be explained by a ceiling effect on consumption. Alternatively, this difference could be explained by the focus on standard chow in the prior experiments, or our use of a different DREADD ligand (Bonaventura et al., 2019), which increased chow consumption at the higher dose even in control rats not expressing DREADD receptors.

We were surprised to find sex-biased effects on consumption, though most prior work demonstrating increased consumption following VP disinhibition was conducted in male subjects (Stratford and Kelley, 1999; Stratford and Wirtshafter, 2013; Covelo et al., 2014). Sex-biased effects could be due to the impact of ovarian and other sex-specific hormones on consumption behavior (Butera, 2010; Xu et al., 2011; Alonso-Caraballo and Ferrario, 2019; Ma et al., 2020). Additionally, the internal hunger state of each animal may differ, and this could be due to sex hormones affecting metabolism and consumption (Butera, 2010; Xu et al., 2011). While female rats in this study weighed less than the male rats at the time of testing, they did not significantly differ in their uncorrected consumption of sucrose under control conditions. Estrogen delivery in ovariectomized female rats causes a significant decrease in food intake when coupled with ghrelin delivery, compared with vehicle and saline controls (Butera, 2010). Estrogen-receptor deletion in most brain regions regulated measures of energy homeostasis, mainly increasing body weight in male and female rats, increasing visceral fat mass and daily food intake in female rats, and decreasing heat production, ambulatory movements, and rearing counts in female rats (Xu et al., 2011). These differences suggest that follow-up experiments controlling for the effects of circulating sex hormones on the metabolism and internal state of the animal would be informative.

### Subregional and cell-type–based heterogeneity

Here, our manipulations were targeted to VP GABA neurons located in mid to midcaudal VP locations on the anteroposterior axis. It is possible that more rostral or more caudal expression of DREADDs would yield different results, based on the respective presence of “coldspots” and “hotspots” in these locations where opioids and orexin agonists have been shown to preferentially impact “liking” and consumption of rewards (Smith and Berridge, 2005, 2007; Ho and Berridge, 2013).

It is also likely that there are multiple VP GABA populations (cell-type specific and projection specific) that affect consumption, with potentially competing roles in behavior. Simultaneous manipulation of these distinct populations may lead to variable results in consumption tasks. VP GABA neurons project to lateral hypothalamus (LH), a canonical feeding center (Stuber and Wise, 2016; Rossi and Stuber, 2018), as well as to other areas that are reciprocally connected with LH (nucleus accumbens, lateral habenula, VTA; Stratford and Wirtshafter, 2013; Root et al., 2015; Stuber and Wise, 2016; Sharpe et al., 2017). Therefore, VP GABA excitation could be impacting the LH in multiple opposing ways. Chemogenetic “disconnection” of VP GABA neurons and LH does not significantly impact reacquisition of alcohol-seeking in rats, whereas disconnection of VP GABA neurons and neurons in the VTA significantly decreases reacquisition of alcohol-seeking (Prasad et al., 2020). Selective activation or inhibition of VP GABA neurons projecting to these regions may yield dissociable results on food consumption.

The VP GABA neuron population is also composed of heterogeneous cell types such as Npas1, somatostatin, and parvalbumin-containing neurons, among others (Knowland et al., 2017; Zhu et al., 2017; Prasad et al., 2020; Pribiag et al., 2021; Morais-Silva et al., 2023). Different VP GABAergic cell-type populations can project to distinct areas of the brain involved in reward-seeking and consumption behavior (Prasad and McNally, 2016; Zhu et al., 2017; Prasad et al., 2020). Prior research indicates there may be different effects of basal forebrain GABA neuronal subtypes on consumption of highly palatable rewards (Zhu et al., 2017). Additionally, VP GABA neurons have a heterogeneous response to reward-paired cues and reward (Stephenson-Jones et al., 2020). Finally, VP GABA parvalbumin neurons may have a distinct role in acquisition of alcohol-seeking behavior (Prasad et al., 2020). Therefore, probing the VP GABA cell types that are involved in food consumption may lead to a greater understanding of the neural circuit mechanisms of consummatory behavior.

### Caveats and future directions

Here, we used DREADDs to activate VP GABA neurons during consumption to examine how activation of VP GABA neurons impacts feeding behavior in *ad libitum* fed male and female rats. We found that activation increased consumption selectively in male rats. While we observed sex-biased effects, we did not assess the estrous cycle, and therefore could not assess how the estrous phase impacted our results. Future work examining the impact of the estrous cycle, or the removal of circulating hormones, could help to better understand this sex difference. We also did not test how different hunger states affect consumption in our paradigm. Additionally, our rats were all tested during the light cycle, which likely influenced basal consumption and could influence the contribution of VP GABA neurons to consummatory behavior. Prior work demonstrating null effects of chemogenetic inhibition or excitation of VP inhibition was conducted during the dark cycle (Li et al., 2021; Farrell et al., 2021). Finally, GABA neurons make up a substantial proportion of VP neurons, and more selective manipulation of VP GABA neurons subpopulations based on projection target or more refined cell types will likely yield additional insights into the mechanisms by which the VP influences food consumption.

## Synthesis

Reviewing Editor: Jibran Khokhar, Western University Department of Anatomy and Cell Biology Decisions are customarily a result of the Reviewing Editor and the peer reviewers coming together and discussing their recommendations until a consensus is reached. When revisions are invited, a fact-based synthesis statement explaining their decision and outlining what is needed to prepare a revision will be listed below. The following reviewer(s) agreed to reveal their identity: Qingchun Tong.

This manuscript, one of the few examining sex differences in VP, describes a study in which VP GABAergic neurons were activated or inhibited to investigate their impact on food intake, finding that activation increased feeding on both chow and sucrose in males but not in females; however, several major and minor concerns need to be addressed.

Firstly, the manuscript lacks crucial information such as the period of food consumption recordings and whether the animals gained weight, making it necessary to analyze the correlation between animal weight and consumption. See https://pubmed.ncbi.nlm.nih.gov/33199503/

Secondly, The results of Gi-DREADD mediated inhibition experiment are difficult to interpret because the present data failed to confirm that the expressed Gi-DREADD really causes the expected inhibition. Without the confirmed inhibition, the data on feeding cannot be used to support any conclusion. Clearly showing the expression pattern of Gi-DREADD across the sections being targeted and verifying with c-Fos expression (for which they may need a control condition known to have more c-Fos in VP) or other method. Thirdly, given the demonstrated sexual dimorphic effects on feeding, the authors need to confirm that DREADD-mediated activation is the same between males and females (expression profiles and the level of activation). Further, the effects of estrogen on this dimorphism could also be explored further. Lastly, the data in Fig. 4 on the potential side effect of the used DREADD ligand should be presented before the data on feeding as this is an important control and needs to be established before the discussion of the main experiments.

Additionally, the manuscript requires minor revisions for clarity and completeness:

#line 36 "said" cue needs attention

#line 38, Parvalbumin neurons should also be included(Knowland et al ~2018 )

#line 47 add references here

#line 50 Regarding reference to Farrell 2021, were the parameters similar i.e food/water deprived, night/day cycle? "In contrast, inhibition of VP GABA neurons fails to significantly reduce non-operant 51 consumption of palatable food (Farrell et al., 2021)."

Methods

#93 Please specify the final volume of virus injected in the animal

#100 it would be good to add why 0.05 to 0.5 mg/ml concentration of JHU37160 was used.

#115 how long after injection was food weighed? Is it 24 hours or 90 mins?

Were the tests conducted in day and night cycle?

Results

Fig 1 C: Since the study reports sex specific differences, it's important to also conduct sex analysis for cfos activity.

Fig 2 C: if analysed females seem to consume less chow than males when saline is injected.

Fig 4: inhibitory or excitatory dreadd?

#247 : DIO-hM2Dq-mCherry. Typo

## Author Response

We thank the reviewers for their constructive comments. We have completed additional data collection and analyses to address reviewer concerns. We also reorganized the presentation of the data to better incorporate the control data. Specific changes are described in response to each comment below.

Major Revisions 1. Firstly, the manuscript lacks crucial information such as the period of food consumption recordings and whether the animals gained weight, making it necessary to analyze the correlation between animal weight and consumption. See https://pubmed.ncbi.nlm.nih.gov/33199503/ Response: Consumption was measured 90 minutes after the injections, and we did not observe any lingering effects on consumption on the following day. Importantly, all manipulations were counterbalanced for order across subjects, so weight gain over time cannot explained any DREADD-related changes in consumption. Adding weight to any of our models did not improve model fit (all AICs and BICs were greater with weight included in the models) and we also did not find any significant effects of weight or interactions with other factors, including when we analyzed males and females separately. We have revised the Results section to include these new analyses.

2. Secondly, The results of Gi-DREADD mediated inhibition experiment are difficult to interpret because the present data failed to confirm that the expressed Gi-DREADD really causes the expected inhibition. Without the confirmed inhibition, the data on feeding cannot be used to support any conclusion. Clearly showing the expression pattern of Gi-DREADD across the sections being targeted and verifying with c-Fos expression (for which they may need a control condition known to have more c-Fos in VP) or other method.

Response: The reviewer raises an important point. To address this question we collected new data using ex vivo electrophysiology. Surprisingly, while we found a trend towards a decrease in spontaneous firing in Gi-expressing cells in comparison to mCherry-expressing cells during the first 5 minutes after J60 application, any effect of the ligand was absent during the next 5 minute bin. These data have been added to Figure 1, and the experiment is described in the Methods and Results sections. Because these results were inconclusive and suggest that J60 acting at Gi DREADD receptors may not effectively inhibit VP GABA neurons, we have removed the Gi behavioral data from the paper. Now that there are just two viral conditions, we have also reorganized the figures to make direct comparisons of Gq and control data more straightforward.

3. Thirdly, given the demonstrated sexual dimorphic effects on feeding, the authors need to confirm that DREADD-mediated activation is the same between males and females (expression profiles and the level of activation). Further, the effects of estrogen on this dimorphism could also be explored further.

Response: We have added additional statistics confirming that the Gq DREADD is effective in both males and females, based on cFos analysis. While the impact of estrogen is an important future question, we feel it is beyond the scope of the current manuscript, especially given the fact that the first three authors are no longer in the lab.

Lastly, the data in Fig. 4 on the potential side effect of the used DREADD ligand should be presented before the data on feeding as this is an important control and needs to be established before the discussion of the main experiments.

Response: We have now reorganized the figures to include the control data for each behavior in the same figure as the Gq data.

Minor Revisions #line 36 "said" cue needs attention Response: We have replaced the word "said" with "these". #line 38, Parvalbumin neurons should also be included(Knowland et al ~2018 ) Response: Given the large overlap between PV neurons and the other major VP cell types (both GABAergic and glutamatergic) listed, it isn't clear that they should be counted as a separate major cell type. We have added the reference (Knowland et al. 2017) to our list of more specific cell-types in the Discussion. #line 47 add references here Response: We have added references to this sentence. #line 50 Regarding reference to Farrell 2021, were the parameters similar i.e food/water deprived, night/day cycle? "In contrast, inhibition of VP GABA neurons fails to significantly reduce non-operant 51 consumption of palatable food (Farrell et al., 2021)." Response: We have clarified in the Introduction that this experiment was conducted in non-restricted rats. We have also added additional discussion of the potential influence of light cycle into the Discussion. #93 Please specify the final volume of virus injected in the animal Response: We have added this information on this line. It is also included earlier in each description of the viral groups. #100 it would be good to add why 0.05 to 0.5 mg/ml concentration of JHU37160 was used.

Response: We have added an additional sentence to the Methods describing our rationale. We also described more recent work from Van Savage and Avegno (2023; published after this data was collected) showing that 1 mg/kg produces anxiety-like behavioral effects, and that 0.5 mg/kg (which produces some non-specific effects in our hands) increases Fos in control rats. #115 how long after injection was food weighed? Is it 24 hours or 90 mins? Response: On observation days food was weighed 90 minutes after rats were placed into the chamber, but rats did not receive any injections on these days. This has been clarified in the manuscript.

Were the tests conducted in day and night cycle? Response: The tests were conducted during the light phase of the cycle. This has been clarified in the manuscript.

Fig 1 C: Since the study reports sex specific differences, it's important to also conduct sex analysis for cfos activity.

Response: As described above, we do not see any interaction between the ligand and sex in our Fos analysis. We have added the following text to the manuscript: "and we observed no interaction between sex and the effect of the ligand (sex by ligand interaction, F(2,6) = 0.12, p = 0.89). Furthermore, when we assessed the impact of J60 in Gq females alone, we found a significant effect of ligand (F(2,3) = 27.59, p = 0.011), despite the null behavioral results reported below." Fig 2 C: if analysed females seem to consume less chow than males when saline is injected.

Response: While females do consume less chow at baseline and on the saline injection day we did not observe any differences in baseline or saline sucrose consumption.

Fig 4: inhibitory or excitatory dreadd? Response: Figure 4 was originally control data, which has now been integrated with DREADD data in the same figures. #247 : DIO-hM2Dq-mCherry. Typo Response: This has been corrected.
